# Degrees of Shortage and Uncovered Ratios for Long-Term Care in Taiwan’s Regions: Evidence from Dynamic DEA

**DOI:** 10.3390/ijerph18020605

**Published:** 2021-01-12

**Authors:** Kuo-Feng Wu, Jin-Li Hu, Hawjeng Chiou

**Affiliations:** 1 Department of Nurse-Midwifery and Women Health, National Taipei University of Nursing and Health Science, Taipei City 112303, Taiwan; kuofeng@ntunhs.edu.tw; 2 Institute of Business and Management, National Yang Ming Chiao Tung University, Taipei City 10044, Taiwan; 3 College of Management, National Taiwan Normal University, No. 162, Section 1, Heping E. Rd., Taipei City 10610, Taiwan; hawjeng@ntnu.edu.tw

**Keywords:** long-term care, data envelopment analysis, degree of shortage, uncovered ratio

## Abstract

The government is facing the country’s aging population and low birth rate have led to a severe shortage of its healthcare workforce in Taiwan after 2003. In order to explore the status of the country’s degree of long-term care shortage and uncovered ratio, this research uses the Push-Pull-Mooring (PPM) theory to explain long-term care efficiency during 2010–2019 in each city and county. We collect longitudinal-sectional data for 2010–2019 from the Ministry of Health and Welfare’s Department of Statistics for 22 administrative regions in Taiwan in each year and employ dynamic data envelopment analysis (DEA) to evaluate the overall technical efficiency and the disaggregate output insufficiency to explain the research results. The main findings are as follows: (1) Cities near the capital Taipei have the highest degree of shortages in long-term caregivers and high uncovered ratios of people who need long-term care. (2) Presently, there is no demand to increase the number of long-term care institutions in Taiwan. (3) The government should introduce new long-term care certificates through national examinations in order to develop a stronger professional workforce in this field.

## 1. Introduction

Taiwan and other parts of East Asia experienced the Severe Acute Respiratory Syndrome (SARS) outbreak in 2003, which led to a sudden and widespread break-out that severely strained health systems. As a result of this epidemic, the medical staff workforce in Taiwan has become strained and insufficient due to a shortage of health care workers and an aging society after 2003. The United Nations World Population Prospects (2019) [1] pointed out that, for the first time in human history, adults over 65 years old outnumbered children under 5 years of age worldwide in 2018; moreover, in 2015, there were more than twice as many elderly persons over 65 years old as children under 5. The proportion of the world’s elderly population is expected to reach nearly 12% in 2030, 16% in 2050, and possibly almost 23% by 2100 [2].

As the elderly population continues to increase in number, countries are investing significantly in long-term care resources. For example, the United States has provided insurance since 1965 under the government-run Medicaid and Medicare programs, which cover home and nursing care (mainly medical care), but strictly speaking, there are no complete or separate long-term care systems in the United States. The U.S. government even reduced Medicare, Medicaid, Children’s Health Insurance Program (CHIP), and marketplace subsidies by about 25%, or $1.1 trillion, in its 2019 budget. Three-fifths of the budget relates to Medicare, which covers 61 million people over the age of 65 or disabled, while the other two-fifths go to Medicaid and CHIP. Social care services produce outcomes that impact several critical aspects of the elderly over the age of 65. From the perspective of such services, the long-term care policy objective is not to spend the least amount of money, but to produce the best quality of life for the elderly. The average active life expectancy (A.L.E.) for men and women was 69.0 and 73.9 years old in Taiwan in 2017, respectively [3], while the number of unhealthy live years for men and women is 8.3 years and 9.8 years. The data indicate in Taiwan that the elderly need long-term care service for an average of 9 years.

Family sizes around the world are mostly getting smaller now. In Spain, an Alzheimer’s disease study of found that the family caregiver is usually a middle-aged woman with medium-level education and living at home. The researchers attributed this women’s intrinsic sense of obligation. These caregivers must modify their lifestyle and habits to fit their new caregiver role. They suffer from long-term emotional distress, and neuropsychiatric symptoms negatively affect their mental health. When the relationship between caregiver and recipient deteriorates, it will also worsen the severity of neuropsychiatric symptoms [4]. In the United States, older caregivers are usually white, and most caregivers are their spouses. They have a college degree and earn an average of US$5500 a month. Caregivers’ higher health satisfaction, well-being, high education level, and high income can enable individuals to help solve health problems and reduce depression. Those conditions can provide support services and offer good care services [5]. In the future, nursing homes could alleviate the elderly care workforce’s problems by increasing the number of long-term care students, professionals, and caregivers in the United States [6]. The situation is similar in Taiwan, there are fewer and fewer family members, and there is a lack of caregiver supply in Taiwan.

Taiwan’s Long Term Care Plan 1.0 was formulated in 2007, and an investment of NT$81.7 billion was initiated during 2017–2016. Subsequently, in September 2016 the Legislative Yuan passed the Long-Term Care Ten-Year Plan 2.0 from 2017 to 2026, for an estimated much larger budget of NT$472.1 billion. This plan will expand the target groups and services when integrating social care, under the goal of creating a comprehensive care system that combines medical care, L.T.C. services, housing, prevention, and social assistance to allow people with disabilities to receive the care they need within a 30-min drive of their home. This program has set up three types of service centers for people needing long-term care. Tier A refers to community-integrated service centers that coordinate and link care service resources according to the care plan designated by the care managers, establishes localized service delivery systems that integrate and connect to Tier B and Tier C resources, and provide pick-up and transport services relating to the three tiers through community transport and care transport personnel. Tier B denotes combined service centers that elevate community capacity, provide L.T.C. services, and increase the public’s diversity of benefits. Tier C covers L.T.C. stations located in the neighborhoods that provide respite services in those areas and implement primary prevention programs.

According to the ten-year long-term care plan of the Ministry of Health and Welfare, at the end of 2018 [7], the number of people needing long-term care services at home was as high as 96,522, followed by 8968 people who needed daytime care, while only 693 needed care at home. The number of cases requiring home services was 56,038 (58%) for women and 44,840 (42%) for men.

As the country’s overall population structure becomes older, the demand for long-term care is increasing rapidly, but the number of nurses and the increasing aging rate cannot balance supply and demand. According to statistics released by the Ministry of Health and Welfare of Taiwan, the average nurse-patient ratio in the world in medical centers is 1:7.7, whereas the nurse-patient ratio in Taiwan is 1:8.6 [8], which is much higher than that in Japan (1:7) [9] and the United States and Australia (1:4) [10]. Therefore, the government’s training of long-term licensors and the setting up of care service stations are alternative solutions for long-term care workforce needs. However, under Taiwan’s long-term care service capacity improvement plan, only 21% of the total number of licensed long-term care employees had received a certificate for completing training in September 2017 [11,12].

Hu et al. [13] used the carrying capacity theory to explore the primary medical staff’s distribution in Taiwan’s administrative regions. Their study found that the supply of medical staff has increased year by year but is concentrated in certain administrative regions and shows overall a shortage of medical staff, more so for nurses than for doctors. The shortage of nurses has pushed Taiwan’s health system to a disadvantageous situation by crowding out caregivers who work in long-term care facilities. Overall, the understaffing of the health care system has become a critical issue in Taiwan.

Taiwan’s problems now include increasing its aging population and the shortages in nurses and long-term care staff. According to the Push-Pull-Mooring theory, caregivers can be regarded as a kind of human capital in each county or city. When the hospital or regional area has enough workforce, it can attract people to move in. The Long-Term-Care Plan 2.0 can help the elderly move forward by preventing incapacitation and utilizing hospice care at home, thus reducing disability and decreasing the number of years requiring long-term care. These goals can only be achieved using outstanding long-term care employees. The resources invested by the government are expected to improve the quality of long-term care. Therefore, this research explores the shortage or surplus of long-term care efficiency spanning a period of ten years in Taiwan.

As of March 2020, the total number of long-term care institutions, nursing homes, and veterans’ homes hit 1649, with a total of 114,308 beds, revealing a bed number that is far below the number of disabled, elderly individuals. There are currently 492,399 elderly disabled persons over 65 [14]. With the gradual aging of the population structure, long-term care and the adequacy of said workers have become national issues. Because of the influence of economic development, the second purpose of this research is to use the Push-Pull-Mooring theory to explain the migration theory and discuss the dynamic DEA efficiency of each city and county for 2010–2019 with regards to long-term care in the country.

## 2. Literature Review

There is shortage of health care workforce throughout the world, and various governments are facing high demand for care due to their aging societies. An alternative mechanism appears to be needed to deal with this medical staff shortage. Patient-centered nursing homes in the U.S. connect elderly adult resources and providers to assess the goals and strategies for taking care of older persons. Nursing homes provide primary health care management, patient rights advice, community communications, and integrated elderly care services. In the future, the problems of the elderly care workforce in U.S. nursing homes could be solved by increasing the number of long-term care students, professionals, and caregivers [6]. Moreover, the application of technology can also reduce contact between people, which is also a way to avoid disease transmission.

With the development of artificial intelligence, many machine learning models have been used in the early study of diabetes risk, such as the novel ensemble learning algorithm, which can investigate personal information, eating habits, exercise status, and family history of the elderly to predict incidences of the disease [15]. More advanced ways of using telemedicine can be done through the predictive diagnosis of artificial intelligence, by collecting data through a structured database via data mining, and from the small collection of patient information on at-home caregivers. Like novel ensemble learning algorithms, artificial intelligence skills can provide different results and help analyze a model to predict patients’ health risks. The results can be used as future hospital resources for home caregivers, hence providing patients at home with better treatment and reducing the risk of in-hospital infection [16]. In 2007, the Taiwanese government began to promote a program of setting up long-term care departments and cultivating talent to improve healthcare workers’ needs. Taiwan has a national health insurance (NHI) system that takes care of residents of all ages, but because it faces an aging population and low birth rate, medical workforce demand has increased.

As Taiwan’s society ages, the demand for elderly healthcare services is rising, especially among middle-aged and older patients who are disabled and living independently. Because of the falling population of children and changing family structure, most families cannot afford long-term care. There are many middle-income and low-income families on the verge of poverty in Taiwan and have a monthly income of less than NT$30,000 (about US$1000). For this reason, they cannot afford long-term care costs and need to take on the responsibility of care by themselves or through family members [17].

The Taiwan government started to promote a long-term care plan in 2007 and launched Long-term Care Plan 2.0 in 2017. Starting in 2018, a long-term care service payment system was activated to promote the establishment of care centers categorized into three tiers: A, B, and C. In May 2019, there were 545 Tier A centers, 3439 Tier B centers, and 2014 Tier C centers [18]. Long-term care resources in all counties and cities across Taiwan have developed rapidly under governmental resources for over ten years. However, due to significant differences in urban and rural areas’ population structures, there are substantial gaps in long-term care services planning and development in different counties and cities.

There are several challenges in the Long-term Care Plan 2.0 implementation process. For example, long-term care workers face cultural and social structure challenges that require more efficient coordination among long-term care managers, attendants, indigenous families, one-step service delivery centers that can integrate different care options, and better indigenous services in the future. Disability studies have found that age significantly affects long-term care services, inpatient services, emergency services, and outpatient services [19].

Demographers have pointed out that migration is a form of changed behavior associated with people’s residence [20]. Three forces influence migration: (1) the push effect, which is a negative factor that leads people to leave their original homes; (2) the pull effect, which is a positive factor that attracts people to their places of residence; and (3) the mooring effect, which refers to personal and social factors that accelerate the departure of migrants or people’s continuation of living in the places of their original homes. These three forces make up the Push-Pull-Mooring (PPM) model, which explores middle-aged and elderly patients’ intentions to switch to cloud healthcare services [21]. Lai and Wang (2015) [22] showed that the elderly understanding cloud services could help solve medical service conditions; for example, changing their behavior toward a dependence network service can resolve the workforce shortage. Using cloud services can improve the elderly’s quality of medical treatment. The push effect refers to pushing away cynical motives—for example, in an online real-person English learning platform and negative feelings in learning convenience, service quality, and perceived price. The pull effect is a significant e-learning motivation in an online real-person English learning platform. The mooring effect demonstrates switching intentions, which incur switching costs. If a customer has become accustomed to an original online platform and wants to change to another forum, then he/she needs to spend money and time to get used to the new user interface [23].

According to the Push-Pull-Mooring theory, long-term care workers in each county or city can be regarded as a kind of human capital. The Human Capital theory is developed based on the value, rarity, imitability, and substitutability of personnel from the Resource-based View (RBV). It can create a competitive advantage for organizations through human capital [24]. Therefore, a company or area with an adequate workforce can attract people to move there. Migration behavior is a human cost input that results in profit maximization. People are expected to move to a county or city when it results in more significant benefits than the moving costs.

For this reason, some people choose to migrate. In health care fields, there is also a migration of talented physicians and health care workers from more impoverished places, leading to a weakening of the health care system and a severe loss of medical talent. Physicians’ push effect includes political instability and corruption, inadequate equipment and facilities, and an inability to support high-quality child-care programs. Physicians’ pull effect includes strong and robust health systems, political stability, and improved quality of life for physicians and their families [25]. The PPM framework has been applied in mobile personal cloud storage services. Moreover, the pull factor’s recovery significantly increases users’ willingness to switch to another technology [26].

Many scholars have used DEA in the medical field to help analyze hospital operating efficiency. The commonly searched outputs in Taiwan’s Ministry of Health and Welfare database include the number of medical practitioners, number of beds, number of general outpatients, number of particular medical users, monthly clinical practice registrations of licensed nurses, number of medical operations, and average number of hospitalized patients. One systematic study of the nursing home literature adopted DEA analysis, finding that 68% of papers employed the CRS model and 86% used input-oriented technical efficiency. For variable selection, 16 articles utilized full-time equivalent staff (including nursing and non-nursing) as the input variable, while a few papers took population aged over 84 as the output variable. In the DEA field of research, most articles used several beds, full-time nurses, and labor wage as inputs and the number of resident days, number of residents, or case-mix index as outputs [27].

Another paper used windows DEA to evaluate maternal health services’ efficiency in different administrative regions of Mexico, finding that the efficiency score is the best in the northern region. The lowest technical efficiency scores are in the south, where there is economic inequality under the presence of indigenous populations. The most impoverished areas have the least efficient maternal health score. For this reason, the Mexico government needs to reorganize the allocation of resources and the criteria for assignment in its administrative regions [28].

Hu and Huang [29] noted that the operating efficiency of public hospitals in Taiwan is deteriorating, and that the efficiency of hospital operations can be improved by the utilization of ward space and by investing in medical equipment and beds. Hu, Chang, and Chung [6] employed population, regional income, local government expenditure on healthcare, physicians, and nurses to explore physicians’ and nurses’ target quantities in Taiwan’s administrative regions. The study found that, except for Taipei City and Chiayi County, the number of medical workers in other cities and counties did not reach the target number, indicating the need to increase the number of medical workers.

Previous literature has used overall technical efficiency or cost efficiency as the basis for creating efficiency reports. However, this approach does not consider the differences in disaggregate input efficiency scores. Therefore, this present paper explores the disaggregate input efficiency score of Hu and Chang [30] using different county variables over ten years.

This research combines data envelopment analysis (DEA) and a PPM framework to discuss long-term care efficiency. It fills this gap in the literature and also takes Taiwan as an example to compute the distribution efficiency of a shortage in long-term care staff and to assess the target quantity of long-term care staff, the number of caregivers, the uncovered ratio of long-term care, and the actual number of residents in long-term care facilities in administrative regions. Findings present the problem of an urban-rural divide along with disparity in the country’s outer islands.

## 3. Methodology

### 3.1. Sample and Data

This study discusses counties’ human capital efficiency and cities’ long-term care institutions from the migration theory perspective. We collect data for 2010 to 2019 from Taiwan’s Longitudinal Study on the Ministry of Health and Welfare’s website. Due to our study’s purpose and the literature review, this research uses non-parametric techniques that observe inputs, outputs, and carry-over variables based on actual data from each county in Taiwan. This research uses the output-oriented dynamic slack-based DEA model proposed by Tone and Tsutsui [31] to compute the efficiency and target output levels.

### 3.2. Variables and Measures

This study’s DEA framework was initially intended to evaluate non-profit organizations’ effectiveness [32], but has since been widely used in various production enterprises and public and private sector organizations. This approach finds an optimal solution to evaluate the relative efficiency of the decision-making unit (DMU), as efficiency represents the most favorable result for the assessed unit under an objective environment.

Charnes et al. [32], as well as Banker et al. [33], further developed various application models of DEA, thereby indicating it is a mature analytical technique at home and abroad. DEA can evaluate multiple outputs and inputs simultaneously and is not affected by units of measurement. The weights in the model are generated by mathematical programming, reducing human judgment error. In a model setting, DEA can be divided into constant returns to scale (CRS) and variable returns to scale (VRS). CRS suppose the relationship between input and output has a fixed return and that one unit of input brings about one unit of output.

There are three primary sources of inter-temporal dependence between input and output. The first source is the effect of capital stock, such as a company’s machinery and equipment, which can improve productivity. When a production cycle spans multiple periods, the change in capital stock or investment capital will lead to inter-period dependence on input and output. Capital stock does not necessarily increase or decrease productivity immediately; instead, a period of adjustment is necessary for the DMU to adapt to new equipment. This study regards the input variables as a kind of capital inventory. The second source is a lagged output. During some production processes, there may be a delay between input and output. For example, nurses or caregivers employed in long-term care facilities must engage in long-term care training before being hired. When they enter the workplace, it takes some time to internalize what they have learned. Delays will occur between input (long-term care training courses) and output (providing good long-term care service quality). The third source is the capital-output. It is impossible to directly measure the intermediate output or capital-output in some production processes, improving future periods’ productivity.

This research uses the regional elder population of over 65 years old as the carry-over variable between two consecutive years. It can be classified as an intermediate output, but not an actual output. In dynamic DEA, input and output can further correlate with the efficiency scores between periods through the dependent linkage variables. Thus, the efficiency scores between periods no longer exist independently.

**Figure 1:**
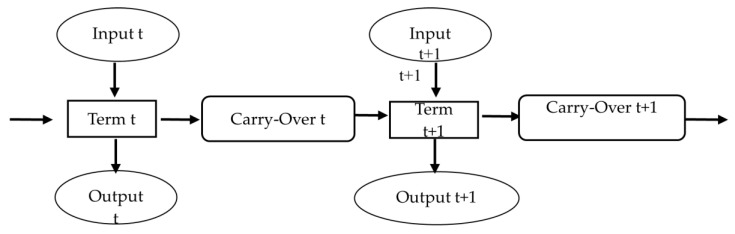
Dynamic Structure.

We conduct dynamic DEA on ten years of data, and the input and output terms of each DMU do not necessarily appear in the same period. Therefore, the traditional DEA model cannot evaluate efficiency; instead, the dynamic DEA model can evaluate the production efficiency. Tone and Tsutsui [31] constructed a dynamic structure, leading to the efficiency of period t and the efficiency of period t+1 no longer being independent (Figure 1). The dependency is developed through the deferred effect. The dynamic DEA model has the following characteristics: (1) it can compare the long-term efficiency of the DMU; (2) the non-radiation SBM model can analyze input and output items independently, and thus non-uniform adjustments are allowed; and (3) between two consecutive years, carry-over variables act as the link. Figure 1 illustrates the relationships among inputs, outputs, and carry-over variables across years.

As shown in Figure 1, each DMU has its input and output in period t, linked to the deferred r variable of period t and period t+1, respectively. Assuming that the time t of the data is from period 1 to period t, a manager notices the continuity from period 1 to period t. The link between t and t+1 is made with a deferred variable, and the desirable good link is defined as the effect desired by the manager when the deferred variables are good. For the DMU, there is a good deferred effect, or it can contribute to a better output result.

This study uses the output-oriented CRS model and assumes that the link is a good carry-over. All weights are set equal to each term. The output-oriented overall efficiency $\tau_{o}^{*}$ is defined by:

(1) $$\frac{1}{\tau_{o}^{*}}=max\frac{1}{T}\overset{T}{\underset{t=1}{\sum }}W^{t}\left[1+\frac{1}{s+ngood}\left(\overset{s}{\underset{i=1}{\sum }}\frac{w_{i}^{+}s_{it}^{+}}{y_{iot}}+\overset{ngood}{\underset{i=1}{\sum }}\frac{s_{it}^{good}}{z_{iot}^{good}}\right)\right]$$

Here, y*_iot_* is the *i*^th^ output of region *o* at time *t*; z*_iot_* is the *i*^th^ carry-over variable of region *o* at time *t*; $w_{i}^{+}$ is the weight to output *i* and satisfies the condition, $\overset{s}{\underset{i=1}{\sum }}w_{i}^{+}=s$; and *T* is the total number of years. In this paper, the weights are all equally assumed to be one. We then define the output-oriented term efficiency $\tau_{ot}^{*}$ by:

(2) $$\tau_{ot}^{*}=\frac{1}{1+\frac{1}{s+ngood}\left(\sum_{i=1}^{s}\frac{w_{i}^{+}s_{iot}^{+*}}{y_{iot}}+\sum_{i=1}^{ngood}\frac{s_{iot}^{good*}}{z_{iot}^{good}}\right)},\;\left(t=1,\ldots ,T\right)$$

The following condition indicates the link flow (carry-over) between terms *t* and *t* + 1:

(3) $$\overset{n}{\underset{j=1}{\sum }}z_{ijt}^{\alpha }\lambda_{j}^{t}=\overset{n}{\underset{j=1}{\sum }}z_{ijt}^{\alpha }\lambda_{j}^{t+1}\left(\forall i;t=1,\;\ldots ,\;T-1\right)$$

Here, *λ_j_^t^* is the peer weight assigned to region *j* at time *t*. The output-oriented overall efficiency during the period $\left(\tau_{o}^{*}\right)$ is the arithmetic mean of the term efficiencies $\tau_{ot}^{*}$ as noted below:

(4) $$\frac{1}{\tau_{o}^{*}}=\frac{1}{T}\overset{T}{\underset{t=1}{\sum }}\frac{w^{t}}{\tau_{ot}^{*}}$$

The overall technical efficiency score is calculated in the previous equation, making it impossible to distinguish further the efficiency brought by each input or output. Hence, this study refers to the method of Hu and Chang [30] to calculate the efficiency score for the number of long-term care receivers (y_1_) and the number of residents in long-term care facilities (y_2_). The DEA model provides the target output of each output or input variable. Moreover, each output’s disaggregate output efficiency can be found by the ratio of target output/actual output between zero (extremely inefficient) and one (fully efficient).

## 4. Data and Results

### 4.1. Data Sources

This study’s longitudinal-sectional data come from the 2010-2019 database of the Department of Statistics, Ministry of Health and Welfare, R.O.C. (Taiwan), with 22 DMUs each year. We measure efficiency using the dynamic DEA technique. The DMUs consist of 22 administrative regions in Taiwan: New Taipei City, Taipei City, Taoyuan City, Taichung City, Tainan City, Kaohsiung City, Yilan County, Hsinchu County, Miaoli County, Changhua County, Nantou County, Yunlin County, Chiayi County, Pingtung County, Taitung County, Hualien County, Penghu County, Keelung City, Hsinchu City, Chiayi City, Kinmen County, and Lienchiang County. Six counties and cities were upgraded to become municipalities of Taiwan in 2014: Taipei City, Kaohsiung City, New Taipei City, Taichung City, Tainan City, and Taoyuan City. This study assumes this action has created a pull effect, because there are more power and resources at the organization, human resource, and financial budget levels than in non-municipalities.

### 4.2. Descriptive Statistics Analysis

This study uses each municipality and county as a decision-making unit and defines x_1_ is the total number of caregivers in long-term care facilities in a county, x_2_ is the total number of licensed caregivers in a county, and y_1_ is the total number of people needing long-term care services in a county. Moreover, y_2_ is the actual total number of people living in a long-term care institution. The carry-over variable is the number of individuals over 65 years of age in each county or city; it does not directly indicate that counties and cities have improved long-term care productivity when calculating their long-term care technical efficiency, but is closely related to the number of nurses and caregivers working in long-term care facilities. Long-term care facilities can provide adequate long-term care services only.

Table 1 lists the descriptive statistics of the input, output, and carry-over variables. This study explains the distribution of human resources in different cities and counties from the perspective of the PPM theory. DEA is a non-parametric method using linear programming, and correlation analysis is used to examine the isotonic relationship in which an output will not decrease with an increase in any input. Before we engage in DEA analysis, the input and output variables should have an isotonic relationship.

Table 2 shows the correlation coefficient between the input and output variables. A positive relation among them indicates that increasing the inputs does not cause the outputs to decrease. Between inputs, the number of caregivers in long-term care facilities has a 0.747 correlation coefficient with the number of caregivers in temporary care facilities. The correlations between inputs and outputs are all non-negative, showing that the isotonicity property is satisfied for all inputs and outputs in the data.

### 4.3. Technical Efficiency

From the Push-Pull-Mooring theory perspective, the better the efficiency is, the greater is the pull force. This research uses the dynamic SBM input-oriented model to explore efficiency in each city and county. To explain more concisely the states of push, pull, and mooring forces in each county and city, we set up the following formula to represent their respective long-term care shortages. The relation between the uncovered ratio (shortage degree) and disaggregate output efficiency is: Uncovered ratio (shortage degree) = 1 − disaggregate output efficiency. Descriptive statistics of overall average inefficiency during 2010–2019 are shown in Table 3.

If the degree of shortage is equal to 0, then the county and city efficiency scores have reached the target number. In other words, the county and the city have a pull effect, which is defined herein as the population migration pull rate. Conversely, if the degree of shortage is greater than 0, then it means that the target number is higher than the actual number, and the county and city need to increase the number of long-term care receivers (y_1_) or the number of residents in long-term care facilities (y_2_). It also implies that the county or city does not have a pull effect, but could have a push effect, spurring people who require long-term care services to move to other counties and cities. This study defines the push effect as the population migration push rate. The number of elderly individuals over age 65 in each county or city is a good carry-over variable and equivalent to the mooring effect in the PPM theory. In other words, personal and social factors may accelerate the departure of migrants or allow them to remain in the places of their original homes. When the proportion of the population above age 65 is higher in a county or city, it is necessary to provide more excellent services for long-term care receivers and increase residents in long-term care facilities.

Table 4 shows overall insufficiency in long-term care for each municipality. The insufficiency score equals one minus the overall efficiency score. Here, Taipei City overall insufficiency is as high as 0.43 and Taoyuan County’s overall insufficiency is 0.42, whereas the average insufficiency in Hsinchu County (under the variable rural) is as high as 0.40. Except for Lienchiang County, there is no shortage of overall efficiency scores in the outer islands. Lienchiang County ‘s overall insufficiency is the highest in Taiwan at 0.50. Over the last ten years, Penghu County ‘s efficiency scores have been perfectly balanced, but this result is different from that in the literature review, which found that six municipalities in Taiwan have more resources than the other counties and cities, indicating that their insufficiency score should be less than those of other counties and cities. One possible reason is that the efficiency provided by dynamic DEA refers to total efficiency, whereas the influence brought by each variable is ignored. In order to explain the causes of insufficiency in long-term care with municipal, rural, and outer islands areas, Table 5 and Table 6 list the concept of disaggregate efficiency scores for further illustration.

### 4.4. Uncovered Ratio for Long-Term Care in Taiwan

Table 5 reports the uncovered ratios of long-term care in Taiwan’s regions during 2010–2019, which further indicates potential expansions to include elderly in long-term care coverage in a region. For the municipality group, the average shortage is 0.39 for Taoyuan County and 0.38 for New Taipei City, indicating that 39% and 38% more elder people need to be covered by long-term care in these two places not being taken care of. In the rural group, Hsinchu County’s and Hsinchu City’s average uncovered ratios are 0.39 and 0.24 for elderly who need long-term care, but who are not being taken care of. In the outer islands, the uncovered ratio of Lienchiang County rises from 0.01 to 0.84 in 2016–2019. The reason is the decline of its elderly population, leading to its unused capacity for long-term care suddenly increasing. Put briefly, the uncovered ratios of long-term care in both municipal and rural regions indicate inefficient results. These areas should increase nursing numbers or long-term caregivers to improve long-term care efficiency.

Table 6 presents the degree of shortage of long-term care facilities in Taiwan’s regions during 2010–2019. In the municipality group, there is almost no shortage in Kaohsiung City. The average degree of shortage for Taoyuan County is 0.31. One possible reason is that residents tend to stay in long-term care facilities in Taipei City and New Taipei City, there has the best medical care system. The elderly chose to live in Taipei and New Taipei rather than Taoyuan, leading to the highest shortage in Taoyuan City in municipality. In the rural group, Keelung City have no shortage in long-term care facilities, whereas Hsinchu City and Changhua County have the highest degree of shortages. One possible reason is that residents of these two counties tend to take care of their family members who do not live in long-term care facilities, causing insufficient results for long-term care facility occupancy. In the outer islands, the average shortage is almost 0, implying that long-term care facilities there exhibit utilization efficiency.

### 4.5. Annual Average Uncovered Ratio and Shortage Degree in Taiwan

**Figure 2:**
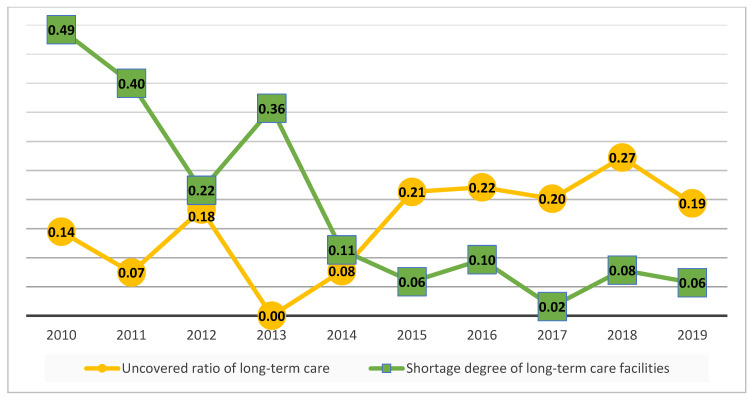
Annual average degree of shortage of long-term care in Taiwan during 2010–2019.

Figure 2 shows that long-term care’s uncovered ratios continue to decline from 0.14 to 0 from 2010 to 2013. However, during 2013–2018 this ratio began to rise. One possible reason might be that the aging ratio of Taiwan is increasing, such that the shortage in the nursing workforce continues to expand. The Taiwan government launched the Long-term Care Plan 2.0 in 2017, and although there was no immediate drop in uncovered ratios of long-term care in 2018, this index did decline in 2019. A lower shortage represents higher long-term care efficiency in the future. The average degree of shortage of long-term facilities is from 0.49 to 0.06 during 2010–2019. However, in 2017 this index fell almost to zero (0.02). In other words, there is virtually no shortage of long-term care facilities in Taiwan on average after 2017.

### 4.6. Discussion

This research employs the Push-Pull-Mooring theory and Human Capital Theory to discuss the shortage efficiency score and further explains the shortage disaggregate efficiency of each city and county in Taiwan in regard to long-term care services. This study adopts the administrative area as the DMU to discuss regional shortage in long-term care. The push effect and pull effect results are used to investigate the uneven allocation of resources among each city and county in Taiwan for 2010–2019.

This research presents the variables for the number of long-term care receivers (y_1_), the actual number of long-term care facilities (y_2_), and how to adjust them to achieve a number that is consistent with the target number. The supply of medical staff increases year by year, but it is concentrated in Taipei City and Chiayi County, showing an unequal distribution of medical staff in Taiwan [13]. While, this research finds that Taoyuan County’s location near Taipei City and New Taipei City, there has the best medical care system. The elderly chose to live in Taipei and New Taipei rather than Taoyuan, leading to the highest shortage in Taoyuan City in municipality. So, there is a more significant long-term care shortage degree in Taoyuan County. It shows that people desire to move to locations with sufficient long-term care resources, leading to presenting a pseudo-thrust result in Taoyuan City. The results are similar to results from the Karan et al. (2016) [25], indicating that political instability and corruption, inadequate equipment and facilities, and an inability to support high-quality child-care programs. Physicians’ pull effect includes strong and robust health systems, political stability, and improved quality of life for physicians and their families. That is likely why physicians move to a better working environment. The same reason the long-term care needs move to Taoyuan. People who receive long-term care will want to be cared for in a county or city with abundant long-term care resources or in a neighborhood community with a sound health care system in order to obtain the best quality of care when needed in an emergency. For this reason, the government should reduce the overall insufficiency by improving the workforce of local caregivers and increasing the number of people who can be admitted to long-term care institutions to achieve a pull result.

About long-term care facilities shortage, Keelung City’s overall insufficiency is almost 0. However, some areas conversely have insufficient resources in the rural group, such as Hsinchu City, it’s shortage is 0.37, As demographic statistics [34] show, in 2019, Hsinchu City had the lowest degree of aging and the highest proportion of supported children under five years old. For this reason, Hsinchu City has too much long-term care facilities, due to the shortage of long-term care facilities in Hsinchu City is the highest in Taiwan.

For the outer islands, the overall insufficiency of Penghu County and Kinmen County is almost 0 from 2010 to 2019. In 2018, the degree of shortage suddenly rose in Lienchiang County, because a decline in the elderly population led to a sudden increase in long-term care’s overall insufficiency. The outer islands are the most resource-poor regions in Taiwan, with no population migration. If long-term caregiver numbers, and number of caregivers in temporary care facilities unchanged, then a decline in the elderly population will lead to a sudden increase in long-term care shortage efficiency, presenting a push effect. Because of the lack of medical care resources in the outer islands, telemedicine is available in the outer islands [16], through telemedicine technology, long-term care patient’s daily health data was collected. When the patient needs emergency medical treatment, such data can provide detailed health information, so that a hospital can immediately receive disease treatment. Cooperation between hospitals and nursing homes may attract people to move to the outer islands, which would also improve long-term care efficiency.

## 5. Concluding Remarks

### 5.1. Conclusions

The analysis of Taiwan’s long-term care workforce leads us to propose the following policy recommendations. (1) Municipalities in Taiwan are generally rich in resources and has a large population, but can experience a severe shortage like its long-term care efficiency score. For this reason, the government should increase the cultivation of a long-term care workforce to reduce the burden on doctors and nurses. (2) The government should also target to increase the number of long-term care workers, because the shortage of long-term care recipients is higher than the shortage of caregivers. (3) Administrative regions have an uneven resource distribution in Taiwan. Counties and cities can aim to reduce long-term care shortages, and the job gap should be reduced between nurses and long-term care workers in order to balance the workload among physicians, nurses, and long-term caregivers. The government should initiate a national license examination for long-term caregivers, which will improve people’s confidence and professionalism in them.

Second, the long-term care shortage degree facilities have gone almost down to 0 in the City or County. One possible reason is that some of the long-term care work is still performed by family members in Taiwan, making the shortage efficiency score of resident capacity reach a critical point of 0.

Third, this research uses the Push-Pull-Mooring theory to explain long-term care efficiency based on two input variables, two output variables, and one good carry-over variable. The push effect and pull effect change population movement, and an essential factor is the mooring effect, which stabilizes such movement. The mooring effect in long-term care depends on a big enough care workforce and good quality of care in the county where the elderly population is currently living. In the future, research may consider adding other variables related to long-term care or selecting other analysis methods to obtain information and thus present implications of long-term care efficiency scores from more aspects. New variables or methods can help provide more information to understand the fundamental reasons for the elderly currently living in a specific county.

Fourth and finally, this study combines the concept of efficiency in production economics with the long-term care research domain. Through dynamic DEA, the efficiency scores are analyzed by the Push-Pull-Mooring theory. When the results show that the DMU has technical efficiency, the long-term care pull effect is observed; when the DMU is inefficient, a long-term care push effect can be found. Between the two effects, the linking variable acts as a form of mooring. We find that the number of people over age 65 in each DMU could be an essential factor in either accelerating migration or keeping individuals in their place of origin. This concept of cross-field integration has not been proposed in previous studies.

#### 5.1.1. Contribution

This study offers several research contributions. First, the concept of cross-field combination has not been proposed in previous studies by bringing together the field of production economics with the clinical care domain. This research suggests such a combination could be incorporated into the theoretical framework of future research. Second, dynamic DEA has never beforehand been applied in longitudinal data analysis of long-term care. This method’s advantage is the inclusion of cost and efficiency concepts, which could provide a more objective result through production economics. Third, DEA does not require parameter estimation and is not limited by a sample number, therefore making it more convenient to use. Fourth, compared with the regression analysis results, the concept of disaggregate input efficiency adopted in this study could further explain how each DMU adjusts the input and output amounts to achieve a high technical efficiency score. In this way, the adjustment direction can be more clearly known. It is hoped that other scholars will apply dynamic DEA to solve performance evaluation-related issues with a time effect in the future.

#### 5.1.2. Limitation and Future Research

This study discusses the shortage of long-term care workforce in counties and cities of Taiwan from 2010 to 2019. All data were collected from the Ministry of Health and Welfare. The results only reflect the shortage of long-term care workforce in counties and cities and not the quality of long-term care in counties and cities, which is the limitation of this study. There are four suggestions for future research. (1) There are many Southeast Asian foreign caregivers in Taiwan, caregiver’s nationality variable can be included in the future research in order to explore the shortages from of the caregiver in Taiwan. (2) Caregivers need to face mental and physical pressure from patients for a long time every day, and their emotions are affected by the patients, thus influencing the quality of care. In the future, researchers might explore the relationship between the mental health status and the quality of care of caregivers. (3) This study only discusses the shortage of human resources. In the future, researchers might compare the income of caregivers who work in nursing homes with those caregivers who are a patient’s family member who does not work, yet live at home and have typical costs they need to pay. Future studies could discuss which kind of care is more economical between hiring caregivers and care from family members. (4) Due to increasing elderly population and the rapid development of information technology in Taiwan, there is a shortage of caregivers in Taiwan, and telemedicine has become more popular. Future long-term care can combine with benefits of telemedicine to solve the long-term care workforce lack problem.

## Figures and Tables

**Table 1:** Input, output, and carry-over variables.

Type	Variable	Min.	Max.	Mean	Stand. Deviation
Inputs	Number of caregivers in long-term care facilities (x_1_)	1	3109	619.25	672.94
	Number of caregivers (x_2_)	4	3566	443.92	506.68
Outputs	Number of long-term care receivers (y_1_)	9	27,082	2445.88	2914.17
	Number of residents in long-term care facilities (y_2_)	9	9029	1582.68	1881.34
Carry-over	Number of people over 65 years old	938	1,142,553	153,055.70	183,880.95

**Table 2:** The correlation coefficients between input and output variables.

Variable	x_1_	x_2_	y_1_	y_2_
Number of caregivers in long-term care facilities (x_1_)	1			
Number of caregivers in temporary care facilities (x_2_)	0.747 **	1		
Number of long-term care receivers (y_1_)	0.613 **	0.909 **	1	
Number of residents in long-term care facilities (y_2_)	0.909 **	0.737 **	0.621 **	1

Note: ** represents the 0.05 significance level.

**Table 3:** Descriptive statistics of overall average inefficiency during 2010–2019.

Statistic	Overall Score	2010	2011	2012	2013	2014	2015	2016	2017	2018	2019
Average	0.27	0.44	0.33	0.21	0.25	0.10	0.16	0.18	0.14	0.21	0.15
St. Dev.	0.14	0.27	0.22	0.18	0.20	0.11	0.15	0.18	0.18	0.19	0.17
Rank		1	2	4	3	10	7	6	9	4	8

**Table 4:** The overall degree of insufficiency for various regions in Taiwan.

Region	Shortage Rank	Overall Insufficiency	2010	2011	2012	2013	2014	2015	2016	2017	2018	2019	Average
Municipality													
New Taipei City	6	0.37	0.58	0.39	0.55	0.31	0.20	0.28	0.35	0.23	0.34	0.16	0.34
Taipei City	2	0.43	0.77	0.56	0.44	0.28	**0.00**	0.24	0.28	0.22	0.28	0.32	0.34
Taoyuan County	3	0.42	0.63	0.49	0.43	0.41	0.23	0.38	0.38	0.37	0.42	0.24	0.40
Taichung City	4	0.41	0.77	0.58	0.29	0.20	0.14	0.29	0.27	0.33	**0.00**	0.07	0.29
Tainan City	10	0.32	0.49	0.51	0.36	0.39	0.08	0.08	0.17	0.21	0.35	0.19	0.28
Kaohsiung City	18	0.16	**0.00**	**0.00**	**0.00**	**0.00**	**0.00**	0.24	0.25	0.27	0.35	0.23	0.13
Rural													
Yilan County	18	0.16	**0.00**	0.22	0.40	0.47	**0.00**	**0.00**	**0.00**	**0.00**	**0.00**	**0.00**	0.11
Hsinchu County	7	0.34	0.43	0.15	0.40	0.45	0.36	0.36	0.36	0.00	0.39	0.31	0.32
Miaoli County	8	0.33	0.53	0.38	0.24	0.54	0.18	0.23	0.19	0.17	0.33	0.21	0.30
Changhua County	13	0.31	0.55	0.44	0.26	0.33	0.18	0.19	0.21	0.00	0.35	0.24	0.28
Nantou County	15	0.23	0.68	0.47	**0.00**	**0.00**	**0.00**	**0.00**	**0.00**	**0.00**	0.06	0.02	0.12
Yunlin County	16	0.19	0.52	0.38	0.22	0.18	**0.00**	0.07	0.08	**0.00**	**0.00**	**0.00**	0.15
Chiayi County	10	0.32	0.55	0.57	0.28	0.38	0.17	0.17	0.12	0.09	0.28	0.07	0.27
Pingtung County	10	0.32	0.63	0.49	0.19	0.42	0.18	0.14	0.05	0.00	0.31	0.12	0.25
Taitung County	17	0.18	0.52	0.45	0.06	0.17	**0.00**	**0.00**	**0.00**	**0.00**	**0.00**	**0.00**	0.12
Hualien County	14	0.24	0.66	0.52	**0.00**	**0.00**	**0.00**	**0.00**	**0.00**	**0.00**	0.03	**0.00**	0.12
Keelung City	20	0.03	0.24	**0.00**	**0.00**	**0.00**	**0.00**	**0.00**	**0.00**	**0.00**	**0.00**	**0.00**	0.02
Hsinchu City	5	0.40	0.51	0.53	0.33	0.63	0.23	0.12	0.30	0.38	0.31	0.31	0.36
Chiayi City	8	0.33	0.73	0.15	0.17	0.22	0.26	0.15	0.31	0.19	0.11	0.14	0.24
Outer Islands													
Penghu County	22	**0.00**	**0.00**	**0.00**	**0.00**	**0.00**	**0.00**	**0.00**	**0.00**	**0.00**	**0.00**	**0.00**	**0.00**
Kinmen County	21	0.01	**0.00**	**0.00**	**0.00**	0.06	**0.00**	**0.00**	**0.00**	**0.00**	**0.00**	**0.00**	0.01
Lienchiang County	1	0.50	**0.00**	**0.00**	**0.00**	**0.00**	0.01	0.54	0.72	0.67	0.61	0.72	0.33
Average		0.27	0.44	0.33	0.21	0.25	0.10	0.16	0.18	0.14	0.21	0.15	0.22

Note: The bold numbers represent the lowest shortage ratios; the underlined numbers represent the highest shortage ratios.

**Table 5:** Uncovered ratios of long-term care in Taiwan’s regions during 2010–2019.

Region	Shortage Rank	Overall Insufficiency	2010	2011	2012	2013	2014	2015	2016	2017	2018	2019	Shortage Average	Shortage Rank
Municipality														
New Taipei City	6	0.37	0.53	0.56	0.44	**0.00**	0.18	0.43	0.49	0.37	0.51	0.28	0.38	4
Taipei City	2	0.43	0.27	**0.00**	0.24	**0.00**	**0.00**	0.36	0.42	0.36	0.44	0.48	0.26	5
Taoyuan County	3	0.42	0.65	0.21	0.39	**0.00**	0.27	0.50	0.47	0.54	0.53	0.38	0.39	1
Taichung City	4	0.41	0.04	**0.00**	0.15	**0.00**	0.16	0.41	0.35	0.49	**0.00**	**0.00**	0.16	11
Tainan City	10	0.32	0.41	**0.00**	0.30	**0.00**	0.04	0.15	0.21	0.35	0.52	0.30	0.23	7
Kaohsiung City	18	0.16	**0.00**	**0.00**	**0.00**	**0.00**	**0.00**	0.39	0.33	0.41	0.52	0.32	0.20	9
Rural														
Yilan County	18	0.16	**0.00**	0.02	0.49	**0.00**	**0.00**	**0.00**	**0.00**	**0.00**	**0.00**	**0.00**	0.05	15
Hsinchu County	7	0.34	0.61	0.26	0.52	**0.00**	0.45	0.51	0.49	0.00	0.54	0.47	0.39	1
Miaoli County	8	0.33	**0.00**	0.38	0.35	**0.00**	0.22	0.32	0.20	0.19	0.42	0.13	0.22	8
Changhua County	13	0.31	**0.00**	**0.00**	0.11	**0.00**	**0.00**	0.15	0.12	0.00	0.43	0.27	0.11	12
Nantou County	15	0.23	**0.00**	**0.00**	**0.00**	**0.00**	**0.00**	**0.00**	**0.00**	**0.00**	0.11	**0.00**	0.01	17
Yunlin County	16	0.19	**0.00**	**0.00**	0.03	**0.00**	**0.00**	0.01	**0.00**	**0.00**	**0.00**	**0.00**	**0.00**	20
Chiayi County	10	0.32	**0.00**	**0.00**	0.24	**0.00**	0.08	0.17	0.05	0.03	0.38	0.13	0.11	12
Pingtung County	10	0.32	**0.00**	**0.00**	0.18	**0.00**	0.17	0.23	0.08	**0.00**	0.44	0.04	0.11	12
Taitung County	17	0.18	**0.00**	**0.00**	0.11	**0.00**	**0.00**	**0.00**	**0.00**	**0.00**	**0.00**	**0.00**	0.01	17
Hualien County	14	0.24	**0.00**	**0.00**	**0.00**	**0.00**	**0.00**	**0.00**	**0.00**	**0.00**	0.06	0.01	0.01	17
Keelung City	20	0.03	0.39	**0.00**	**0.00**	**0.00**	**0.00**	**0.00**	**0.00**	**0.00**	**0.00**	**0.00**	0.04	16
Hsinchu City	5	0.40	0.29	0.03	0.33	**0.00**	**0.00**	0.11	0.35	0.55	0.36	0.37	0.24	6
Chiayi City	8	0.33	**0.00**	0.18	0.14	**0.00**	0.12	0.23	0.47	0.33	**0.00**	0.24	0.17	10
Outer Islands														
Penghu County	22	0.00	**0.00**	**0.00**	**0.00**	**0.00**	**0.00**	**0.00**	**0.00**	**0.00**	**0.00**	**0.00**	**0.00**	20
Kinmen County	21	0.01	**0.00**	**0.00**	**0.00**	**0.00**	**0.00**	**0.00**	**0.00**	**0.00**	**0.00**	**0.00**	**0.00**	20
Lienchiang County	1	0.50	**0.00**	**0.00**	**0.00**	**0.00**	0.01	0.70	0.84	0.80	0.73	0.84	0.39	1
Average		0.27	0.14	0.07	0.18	**0.00**	0.08	0.21	0.22	0.20	0.27	0.19	0.16	

Note: The bold numbers represent the lowest shortage ratios; the underlined numbers represent the highest shortage ratios.

**Table 6:** Degree of the shortage of long-term care facilities in Taiwan’s regions during 2010–2019.

Region	Shortage Rank	Overall Insufficiency	2010	2011	2012	2013	2014	2015	2016	2017	2018	2019	Shortage Average	Shortage Rank
Municipality														
New Taipei City	6	0.37	0.62	**0.00**	0.63	0.47	0.21	0.04	0.08	**0.00**	**0.00**	**0.00**	0.21	11
Taipei City	2	0.43	0.86	0.72	0.55	0.44	**0.00**	0.04	0.06	**0.00**	0.01	**0.00**	0.27	8
Taoyuan County	3	0.42	0.61	0.62	0.46	0.58	0.18	0.18	0.26	**0.00**	0.22	0.03	0.31	4
Taichung City	4	0.41	0.87	0.73	0.40	0.33	0.11	0.13	0.16	**0.00**	**0.00**	0.13	0.29	5
Tainan City	10	0.32	0.56	0.68	0.41	0.57	0.12	0.00	0.13	**0.00**	**0.00**	0.03	0.25	9
Kaohsiung City	18	0.16	**0.00**	**0.00**	**0.00**	**0.00**	**0.00**	**0.00**	0.16	0.04	**0.00**	0.10	0.03	18
Rural														
Yilan County	18	0.16	**0.00**	0.35	0.29	0.64	**0.00**	**0.00**	**0.00**	**0.00**	**0.00**	**0.00**	0.13	16
Hsinchu County	7	0.34	**0.00**	**0.00**	0.19	0.62	0.23	0.05	0.14	0.00	0.08	**0.00**	0.13	16
Miaoli County	8	0.33	0.69	0.39	0.07	0.70	0.15	0.12	0.18	0.16	0.19	0.28	0.29	5
Changhua County	13	0.31	0.71	0.61	0.37	0.49	0.31	0.23	0.29	0.00	0.26	0.21	0.35	2
Nantou County	15	0.23	0.81	0.64	**0.00**	**0.00**	**0.00**	**0.00**	**0.00**	**0.00**	**0.00**	0.04	0.15	14
Yunlin County	16	0.19	0.68	0.55	0.35	0.31	0.00	0.12	0.15	**0.00**	**0.00**	**0.00**	0.22	10
Chiayi County	10	0.32	0.71	0.73	0.31	0.55	0.25	0.17	0.18	0.15	0.16	**0.00**	0.32	3
Pingtung County	10	0.32	0.78	0.66	0.20	0.59	0.19	0.03	0.02	0.00	0.11	0.19	0.28	7
Taitung County	17	0.18	0.68	0.62	**0.00**	0.28	**0.00**	**0.00**	**0.00**	**0.00**	**0.00**	**0.00**	0.16	13
Hualien County	14	0.24	0.80	0.69	**0.00**	**0.00**	**0.00**	**0.00**	**0.00**	**0.00**	**0.00**	**0.00**	0.15	14
Keelung City	20	0.03	**0.00**	**0.00**	**0.00**	**0.00**	**0.00**	**0.00**	**0.00**	**0.00**	**0.00**	**0.00**	**0.00**	21
Hsinchu City	5	0.40	0.62	0.69	0.33	0.77	0.37	0.13	0.25	0.00	0.26	0.25	0.37	1
Chiayi City	8	0.33	0.84	0.12	0.20	0.37	0.36	0.04	0.01	**0.00**	0.20	**0.00**	0.21	11
Outer Islands														
Penghu County	22	0.00	**0.00**	**0.00**	**0.00**	**0.00**	**0.00**	**0.00**	**0.00**	**0.00**	**0.00**	**0.00**	**0.00**	21
Kinmen County	21	0.01	**0.00**	**0.00**	**0.00**	0.12	**0.00**	**0.00**	**0.00**	**0.00**	**0.00**	**0.00**	0.01	20
Lienchiang County	1	0.50	**0.00**	**0.00**	**0.00**	**0.00**	**0.00**	**0.00**	0.06	**0.00**	0.25	**0.00**	0.03	18
Average		0.27	0.49	0.40	0.22	0.36	0.11	0.06	0.10	0.02	0.08	0.06	0.19	

Note: The bold numbers represent the lowest shortage ratios; the underlined numbers represent the highest shortage ratios.
